# Atomic interaction mechanism for designing the interface of W/Zr-based bulk metallic glass composites

**DOI:** 10.1038/srep08967

**Published:** 2015-03-11

**Authors:** Z. K. Li, H. M. Fu, P. F. Sha, Z. W. Zhu, A. M. Wang, H. Li, H. W. Zhang, H. F. Zhang, Z. Q. Hu

**Affiliations:** 1 Shenyang National Laboratory for Materials Science, Institute of Metal Research, Chinese Academy of Sciences, Shenyang. 110016, China; 2 University of Chinese Academy of Sciences, Beijing. 100049, China

## Abstract

The interaction between active element Zr and W damages the W fibers and the interface and decreases the mechanical properties, especially the tensile strength of the W fibers reinforced Zr-based bulk metallic glass composites (BMGCs). From the viewpoint of atomic interaction, the W-Zr interaction can be restrained by adding minor elements that have stronger interaction with W into the alloy. The calculation about atomic interaction energy indicates that Ta and Nb preferred to segregate on the W substrate surface. Sessile drop experiment proves the prediction and corresponding *in-situ* coating appears at the interface. Besides, the atomic interaction mechanism was proven to be effective in many other systems by the sessile drop technique. Considering the interfacial morphology, Nb was added into the alloy to fabricate W/Zr-based BMGCs. As expected, the Nb addition effectively suppressed the W-Zr reaction and damage to W fibers. Both the compressive and tensile properties are improved obviously.

It is known that bulk metallic glasses (BMGs) possess outstanding properties such as high fracture strength and hardness, large elastic elongation limit and excellent wear and corrosion resistance, etc.^1^,^2^,^3^,^4^. However, a majority of BMGs fracture catastrophically in a shear mode under tension or compression at room temperature because of the rapid propagation of localized shear bands^5^, which greatly limits the widespread applications of BMGs as structural materials^6^. In order to improve the mechanical properties, bulk metallic glass composites (BMGCs) containing *ex-* and *in-situ* ductile reinforcements have been proven to be a very effective approach. Various composites increase the ultimate strength and the plastic strain under compression greatly, which puts forward BMGs' applications to a new stage. Among them, Zr-based BMGCs reinforced with W balls^7^, W fibers^8^,^9^ or porous W^10^ have been developed and attract much attention. In the early studies, the compressive strength of 80% V_f_ W fiber (with the diameter of 0.25 μm) reinforced Zr_41.25_Ti_13.75_Cu_12.5_Ni_10_Be_22.5_ (Vitreloy 1) metallic glass matrix composites was about 2250 MPa^9^,^11^. After years of research, the compressive strength increased to 2550 MPa^8^. At the same time, the compressive plastic strain increased from less than 10% to 23% significantly. Furthermore, our previous work showed that both the compressive strength and the plastic strain increased greatly with the decreasing of the W fiber diameter and similar results were shown in many other reports^12^,^13^.

Excellent compressive properties make W fiber reinforced Zr-based BMGCs a very promising material. Greatly improved penetrating performance compared with the traditional W alloys^11^,^14^ captures scientists' interest. For engineering application, however, tensile property is another important factor to focus on. Though the ultimate tensile strength increased from about 1000 MPa^9^ to 1685 MPa^8^ after continuous investigation, it was still much lower than the theoretical value calculated by the rule of mixture. The strengthes of the W fiber of 0.3 μm and the Vitreloy 1 alloy are 2200 MPa^15^ and about 1900 MPa^7^,^9^, respectively, and the calculated strength of the corresponding composite is about 2140 MPa. The difference between the theoretical and experimental strength also exists in many other reports^16^,^17^,^18^. This means the compositing procedure weakens the composite's mechanical property, which becomes the main obstacle of the application of BMGCs. The reasons for the gap reported before are the residual tensile stress in the amorphous matrix formed during solidification and the stress concentration at the interface caused by shear bands or cracks at the matrix^17^. Besides, the interdiffusion and further interfacial reaction between W fibers and active elements in the matrix, such as Zr and Ti, reduce the composite's mechanical properties significantly. Therefore, the interface control is the key point to improve the tensile property of the composites. Unfortunately, for the W/Zr-based BMGCs, more work focus on the types of the reinforcement and matrix, and little work on the interface of composites. A series of high-properties W/Zr-based BMGCs were reported without considering the interfacial characteristics.

It is widely accepted that the most efficient measure to control the interface is to coat the W fiber. The coating not only acts as a transition layer to alleviate the residual stress and the stress concentration, but also works as a protective coating to prevent the fiber from being damaged by the active elements. So far, coating on W fiber is realized by introducing external materials on the fiber surface. However, it is very complicated, difficult and expensive to coat large amount of tiny W fibers with uniform thin layer, especially for engineering application. Besides, coated W fiber is actually a kind of *ex-situ* composite. Just like the BMGCs, the interface control is still the barrier, which is shown in many previous reports^19^,^20^. The interfacial bonding state and the microcracks in the coating as well as the resulting stress concentration are still difficult to deal with. The interaction between W fiber and coating material needs to be considered as well^21^. High temperature is usually needed to obtain strong interfacial bonding, which decreases the mechanical properties of the W fibers to large extent. In addition, the composites are fabricated using melt infiltration process, during which the uneven dissolution and diffusion of coating material into the amorphous matrix easily results in the reduction of the glass forming ability (GFA) of the matrix. Therefore, it is not appreciate to fabricate composites with precoated W fibers.

As mentioned above, composites containing *ex-* and *in-situ* reinforcements have been studied widely, and the major advantage of *in-situ* composite is better interfacial characteristics. Based on this fact, this paper concentrates on creating an *in-situ* coating on the W fiber surface during the infiltration process. The generation of the *in-situ* coating can buffer the stress near the interface and modify the interfacial bonding state. Therefore, we try to search an element possessing higher affinity with W compared with the active elements in the amorphous matrix, and then adding this element into the matrix. Due to stronger interaction with W, the coating element will segregate on the W fiber surface preferentially during the infiltration process and form an *in-situ* coating spontaneously. In addition, minor residue of the coating element in the matrix is inevitable, which should not affect the GFA of the matrix obviously. The structure of *ex-situ* composite combined with *in-situ* coating is expected to show more excellent mechanical properties.

To search the coating element, an atomic interaction model was introduced. By calculating the atomic interaction energy between various elements (such as Nb, Ag, Fe, Co, Ti, Ta and Cr) and W, appropriate interfacial coating element was found out. Sessile drop experiments were carried out to verify the validity of the theoretical prediction. At last, W fiber reinforced Zr-based BMGCs with expected interfacial characteristics and enhanced mechanical properties were fabricated.

## Results and discussion

As discussed before, the decrease of the composites' mechanical properties is mainly attributed to the interaction between W and active element Zr. Inappropriate infiltration process easily results in the interfacial reaction and erosion to the W fiber. In Zr-based alloys or Zr-containing Cu-based alloys, the strong interaction between Zr and W always produces the W-Zr phase at the interface^18^. From the viewpoint of atomic interaction, the elements possessing stronger interaction with W need to be found out. In an *A-B* binary system, according to the “nearest neighbor” interaction model, the change of energy when *A-B* pairs are formed from *A-A* and *B-B* pairs is^22^: 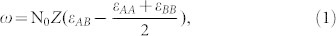
where N_0_ is the Avogadro's number, *Z* is the mean coordination number in the liquid and *ε_ij_* is the interaction energy of an *i-j* pair, which is always negative and lower negative value means stronger interaction. In addition, the heat of evaporation of pure metal *i* is approximately: 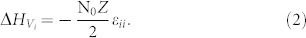


If the molar atomic interaction energy is defined as: 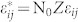, then equations (1) and (2) can be written as: 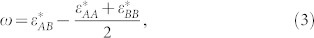

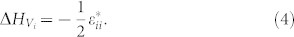


In an *A-B* binary regular solution, the enthalpy of mixing is given by: 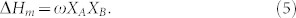


Therefore, the molar enthalpy of mixing of equimolar *A* and *B* is: 
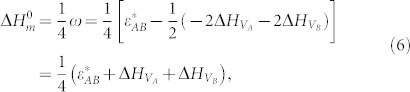
so
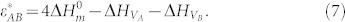


The values of 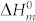 can be calculated by Miedema's model^23^. According to equation (7), atomic interaction energy between various elements can be calculated and the strength of atomic interaction can be characterized by 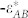. The higher the value of 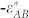 is, the stronger interaction between *A* element and *B* element is.

**Figure 1:**
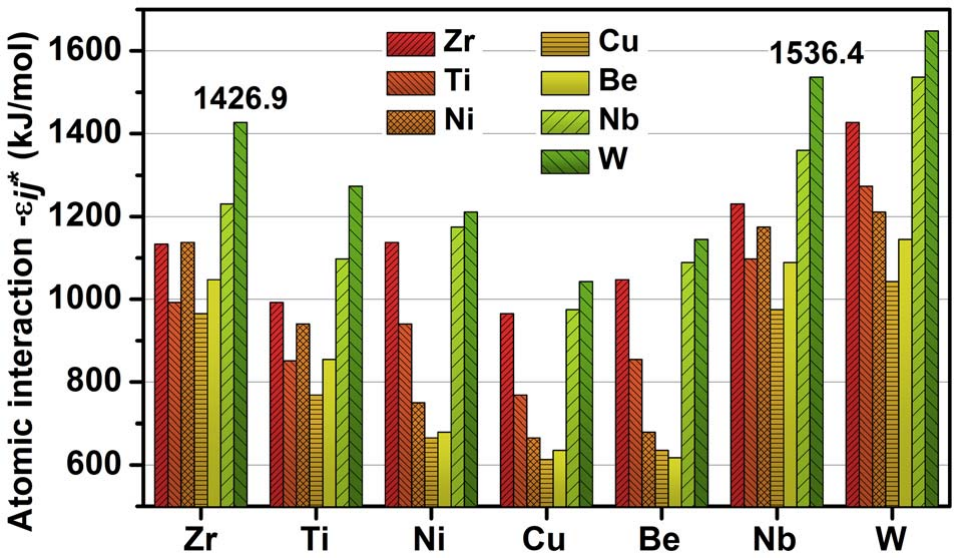
The calculated atomic interaction (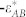) between elements.

Considering the effect of element addition on the GFA of the matrix, the Zr_40.08_Ti_13.30_Cu_11.84_Ni_10.07_Be_24.71_ alloy (denoted as V) with high GFA and strong composition deviation tolerance was selected as the amorphous matrix. The impact of Nb addition was evaluated first. Figure 1 shows the calculated atomic interaction (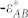) between compositional elements in the alloy and W element. It is obvious that the interactions between W and compositional elements are stronger than those between themselves. The W-Nb interaction is the strongest (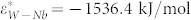) followed by W-Zr interaction (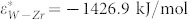, 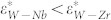). This indicates that W-Zr compound will form preferentially at the interface in Nb-free W/Zr-based alloy composite. Previous research agrees well with the calculation^18^,^24^,^25^. On the other hand, it can be predicted that Nb will segregate on the W surface in Nb-containing system and restrain the reaction between W and Zr.

Similarly, the atomic interaction energies between many other elements and W element were calculated. The results show that the atomic interaction between W and Ta is stronger than that between W and Zr (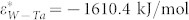). The effect of Ta addition on the interfacial characteristics should be similar to that of Nb addition. In addition, the Ta-Ta interaction is very strong (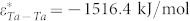). This indicates that Ta atoms in the melt tend to attract each other. So in this work, 5 at.% Nb and Ta are added into the Zr-based alloy respectively (ie. (Zr_40.08_Ti_13.30_Cu_11.84_Ni_10.07_Be_24.71_)_95_Nb_5_ and (Zr_40.08_Ti_13.30_Cu_11.84_Ni_10.07_Be_24.71_)_95_Ta_5_, denoted as VNb5 and VTa5, respectively) to investigate the validity of the prediction.

**Figure 2:**
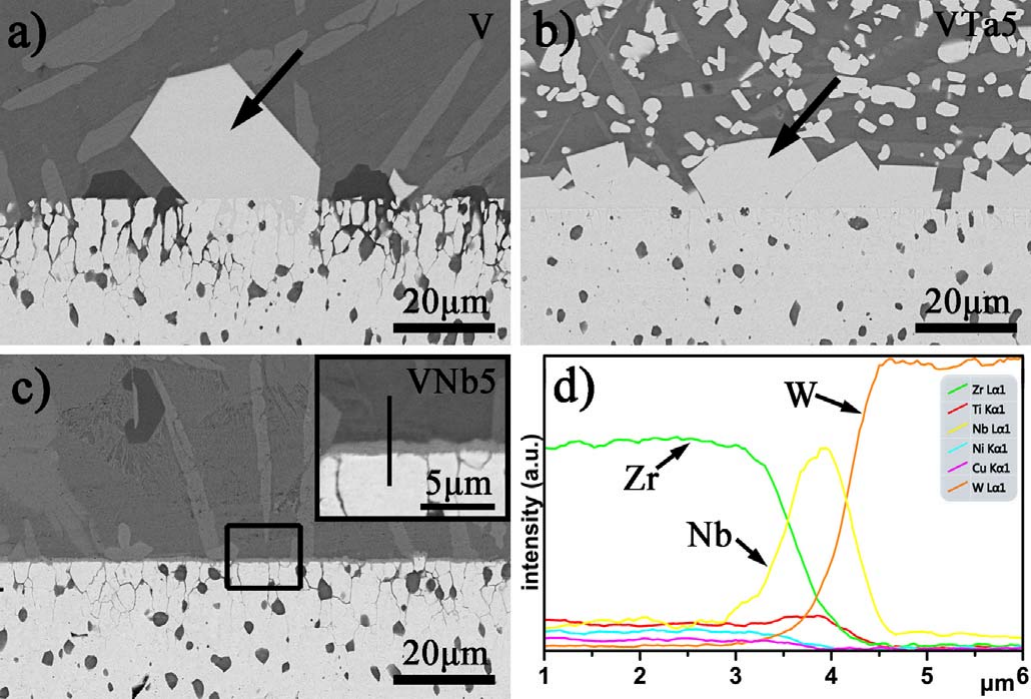
Interfacial SEM images of (a) V, (b) VTa5 and (c) VNb5 alloys on W substrates. The inset is the enlarged view of the interface. (d) EDS scanning along the black line from top to bottom in the inset of (c).

Given the advantage on interface study, sessile drop method^26^,^27^,^28^ was introduced to research the effect of elements addition. The differential scanning calorimetry (DSC) results of V, VNb5 and VTa5 alloys indicates that the liquidus temperatures are 990 ± 5 K. Therefore, the alloys were heated to 1273 K on W substrates and held for 30 min. The morphology of the cross section perpendicular to the interface is shown in Figure 2. Severe reaction occurs at the interface in the original W/V alloy system (Figure 2(a)). The EDS analysis shows that the irregular reaction product (indicated by the black arrows) is W-Zr phase. The surface of the W substrate is eroded obviously and numerous wide erosion ditches emerge on the W surface. This agrees well with the discussion before that active element Zr causes great damage to W fiber and composite's interface.

Figure 2(b) shows the interfacial characteristics of W/VTa5 alloy system. The addition of Ta element changes the interfacial morphology obviously. Bulk product (indicated by the black arrows) appears at the interface, which is proven to be W-Ta phase by EDS. A layer of nearly ellipsoidal particles indicated to be Ta by EDS dispersively distribute in the matrix near the interface. Besides, there is only small and narrow erosion ditches on the W substrate surface. This agrees well with the calculated result before. The strong atomic interaction between W and Ta results in the segregation of Ta at the interface. The W-Ta phase covering the substrate prevents the W from continuous dissolution and reaction with Ta in the matrix. Finally due to the strong Ta-Ta interaction, enriched Ta atoms interact and precipitate from the matrix.

When 5 at.% Nb is introduced, the interfacial reaction is suppressed greatly (Figure 2(c)). There is no obvious reaction product at the interface in macroscopic view. The erosion to the substrate surface is also much weaker than that without element addition. From the enlarged view of the interface, a thin layer of about 1 μm appears at the interface, which is detected to be Nb by EDS. Figure 2(d) shows the EDS linear scanning at the interface from matrix to W substrate. An obvious Nb layer covers the substrate, and the *in-situ* coating is exactly what is expected. Therefore, it can be concluded that Nb segregates at the interface and inhibits the reaction between W and Zr, which further evidences the validity of the atomic interaction mechanism.

In addition to Ta and Nb, the effects of many other elements (such as Ag, Fe, Co, Ti and Cr) were also calculated. As expected, all the experimental results are in accordance with the theoretical calculation. The segregated elements in W/Zr-based alloys as well as some W/Cu-based alloys systems from previous reports^18^,^25^,^29^,^30^,^31^,^32^,^33^,^34^,^35^ are summarized in Table 1. The experimental results (superscript a in Table 1) are shown in Figure S1 in the supplementary file. All the results show that the element with the strongest interaction with W segregates at the interface firstly. The atomic interaction mechanism shows universal adaptability in W/Zr-based alloys systems and can be introduced to guide the design of composite with improved interface.

**Figure 3:**
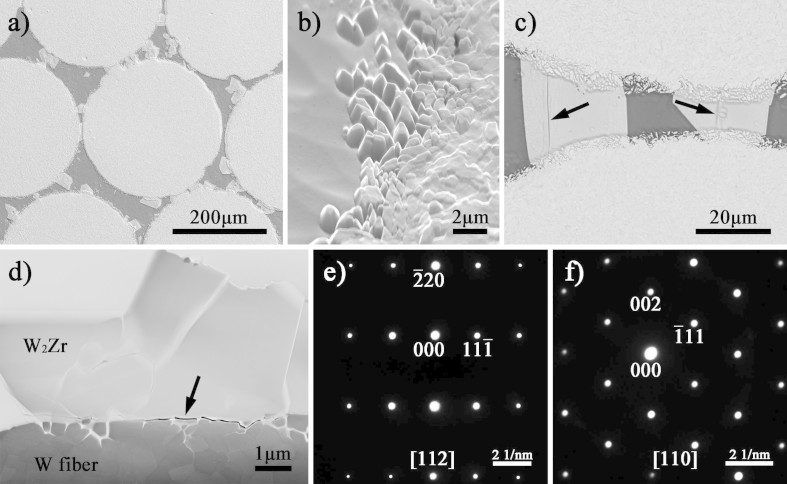
Interfacial characteristics of W fiber reinforced V alloy composite: (a) SEM image of the cross section, (b) the erosion of the W fiber surface, (c) reaction product between the W fibers, black arrows indicate the cracks, (d) HAADF image at the interface, (e) and (f) SAED patterns along [112] and [110] zone axes of the reaction product.

Both Ta and Nb addition improve the interface effectively. However, the addition of Ta results in the formation of bulk products at the interface. The process of preparing BMGCs through rapid solidification inevitably results in strong thermal residual stress, which easily induces the formation of microcracks around the bulk reaction products. Besides, the stress concentration under loading due to the elastic mismatch will also induce the appearance of cracks in early stage. Therefore, the Ta addition exert slightly on the improvement of the composite's mechanical properties. It is also evidenced by compression test. In comparison, the Nb addition generates a Nb rich layer, which can reduce the stress concentration effectively. So in this work, Nb is added into the alloy to investigate the effect of the *in-situ* coating structure on the mechanical properties of the composites. W fiber reinforced V and VNb5 alloys composites are fabricated using infiltration method at 1273 K and 30 min. Figure 3 shows the microstructure of the W/V composites. Severe interfacial reaction occurs and a large number of reaction products appear near the W fiber surface (Figure 3(a)). Figure 3(b) shows the enlarged view of the interface. The W fiber surface is eroded severely even in the area without interfacial reaction, which will decrease the mechanical properties of the W fiber significantly. The peeling of the W fiber surface will result in the continuous dissolution of the inner part of the fiber, which decreases the GFA of the amorphous matrix greatly. Besides, the contact area between W fiber and matrix increases after surface peeling, which aggravates the interfacial reaction. The brittle reaction product easily results in the stress concentration and rapid propagation of cracks under loading. Actually, there exist lots of cracks in the reaction product as indicated by the black arrows in Figure 3(c). The high angle annular dark field (HAADF) image (Figure 3(d)) shows the crack along the interface, indicating strong thermal residual stress and weak adhesion in the composite. Selected area electron diffraction (SAED) result (Figure 3(e) and (f)) indicates the reaction product is W_2_Zr intermetallic (space group 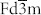, a = 0.7616 nm).

**Figure 4:**
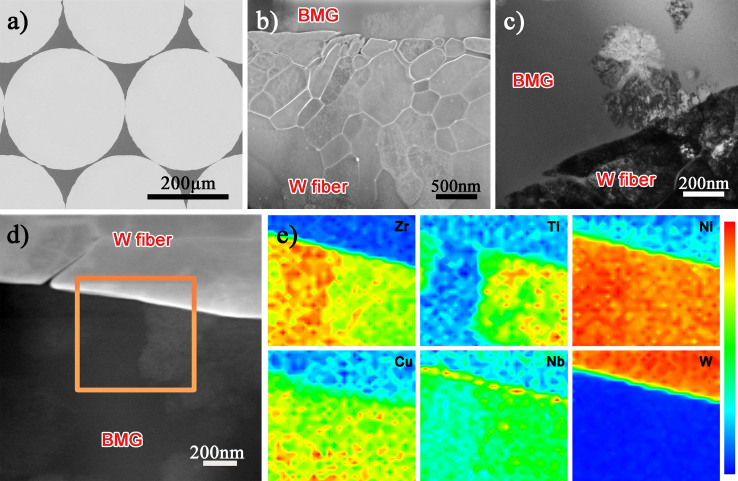
Interfacial characteristics of W fiber reinforced VNb5 alloy composite: (a) SEM image of the cross section, (b) HAADF image of the W fiber surface, (c) TEM bright field image of the interface, (d) HAADF image and (e) EDS mapping of the corresponding area in (d).

Figure 4 shows the interfacial characteristics of W/VNb5 composites. 5 at.% Nb addition restrains the interfacial reaction obviously and the fiber surface keeps intact in macroscopic view (Figure 4(a)). Figure 4(b) shows the HAADF image of the W fiber surface. The erosion depth is about 0.5 μm, which is much shallower than that in W/V composite. Besides, some crystals appear in the matrix, which is also shown in Figure 4(c). The reason for this should be the decrease of the GFA of the matrix. Firstly, the dissolution of W fibers affects the GFA to a certain extent. More importantly, the outer stainless steel tube will dissolve severely under high temperature and long holding time. The EDS mapping (Figure 4(e)) of the corresponding area in Figure 4(d) indicates that the crystals are rich in Nb and Ti elements. Furthermore, an obvious Nb rich layer similar to but much thinner than that in Figure 2(c) appears at the interface. The contact angle of the sessile drop experiment is measured to be 29°, and the interface density (defined as the ratio of interfacial area to alloy volume) is calculated to be 3.23 ± 0.01 mm^−1^, while that in the composite with close-packed W fibers is up to 129.88 mm^−1^. According to the calculation of the interface density, the thickness of the Nb coating in the composite is about 25 nm.

**Figure 5:**
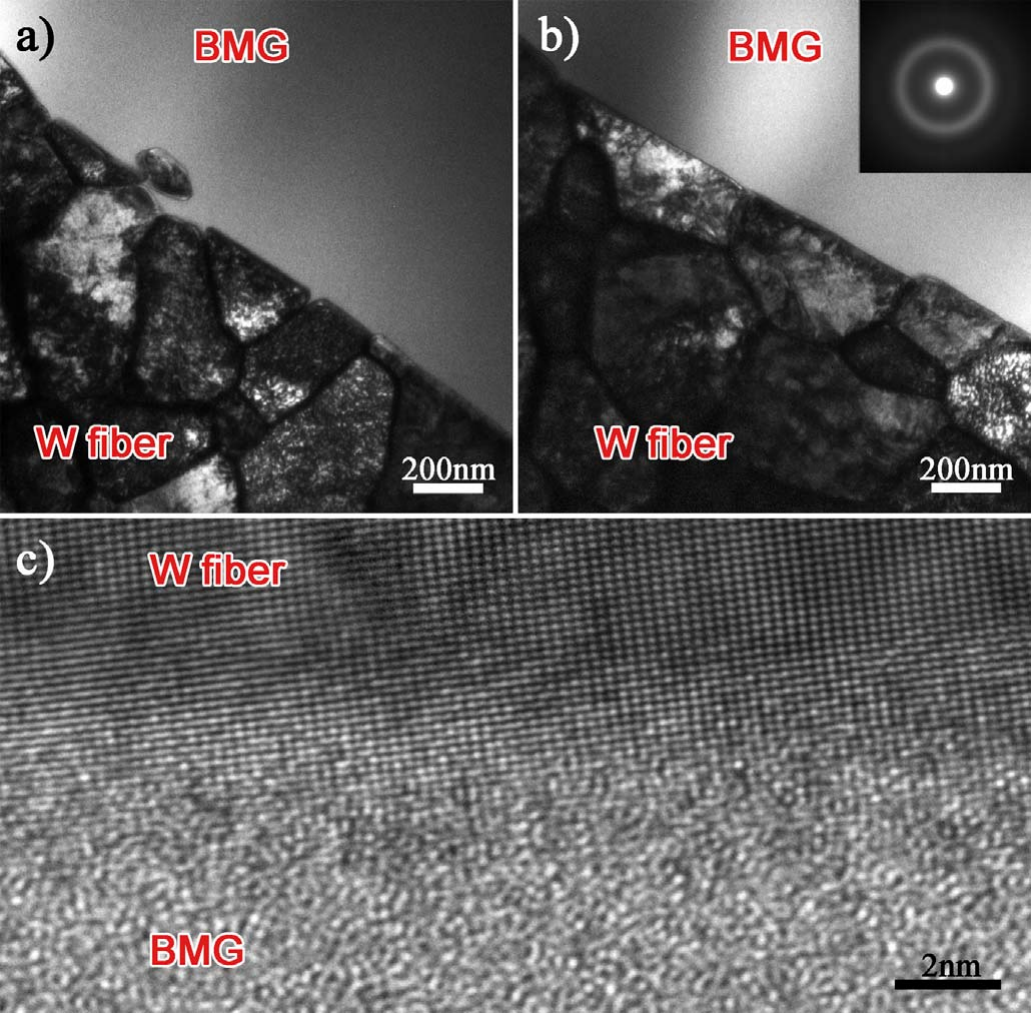
Interfacial characteristics of (a) W/V and (b) W/VNb5 composites, the inset shows the SAED pattern of the amorphous matrix, (c) High resolution TEM (HRTEM) image of the interface in W/VNb5 composite.

To prevent the formation of the crystals in the matrix and reduce the dissolution of the W fibers and the outer tube, lower infiltration temperature and shorter holding time (1123 K and 5 min) were carried out to fabricate the composite. Besides, alloys with different Nb contents were used as matrix. Figure 5 shows the microstructure of the composites. TEM bright field image (Figure 5(a)) shows slight intergranular erosion on the W fiber surface in the composite without Nb addition. While in the W/VNb5 composite, the fiber surface keeps smooth and flat, and the matrix is amorphous (Figure 5(b)). Besides, the high resolution TEM (HRTEM) image (Figure 5(c)) shows perfect interfacial bonding, which is expected to improve the mechanical properties of the interface and the composite.

**Figure 6:**
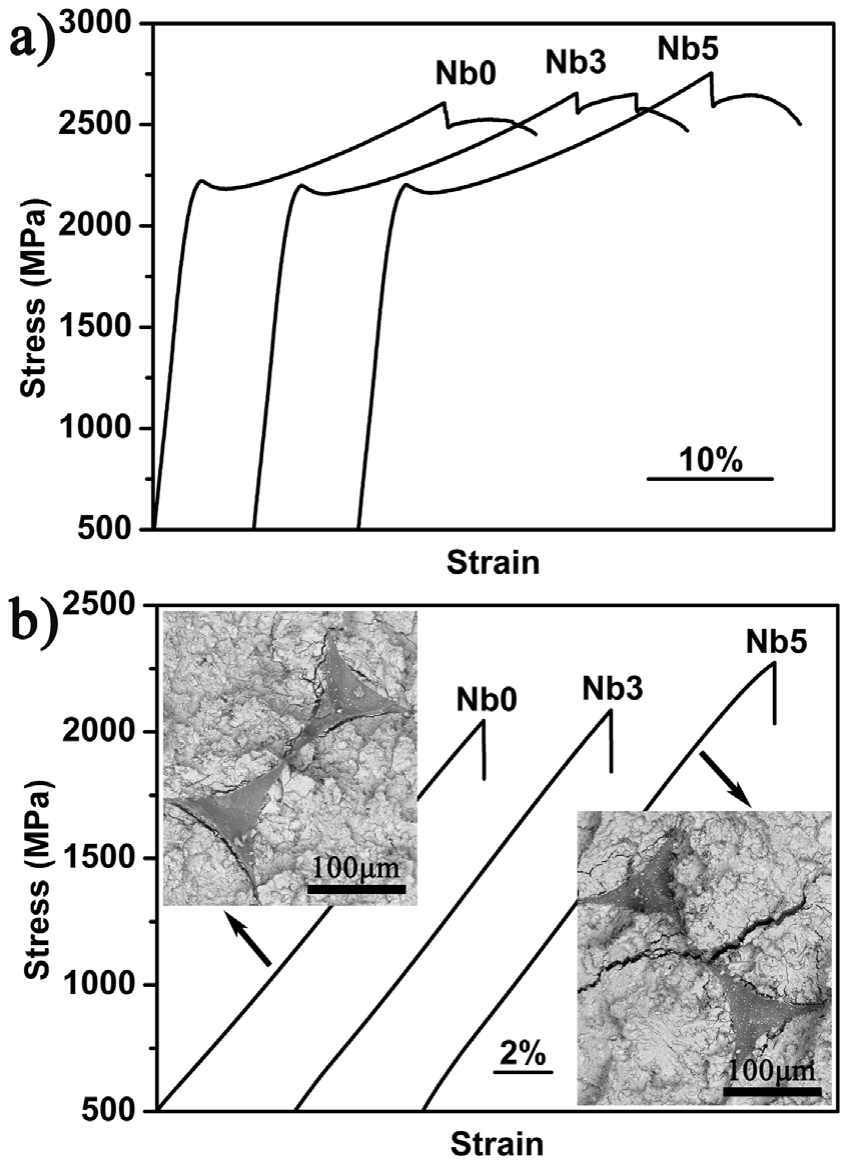
(a) Compressive and (b) tensile curves of BMGCs with different Nb contents. The insets show the fracture surface of corresponding composites under tension.

Figure 6 shows typical engineering stress-strain curves of three composites under compression and tension, respectively. Both the compressive and tensile properties increase greatly with the Nb addition. The compressive strength of W/V composite is about 2600 MPa, while that of W/VNb5 composite is up to about 2750 MPa accompanied with improved strain plasticity. More importantly, the tensile strength increases from about 2040 MPa to 2270 MPa or so, which is the highest among this kind of composites. Nb addition increases the tensile property to the same range of the theoretical value calculated by the rule of mixture. This is directly related to the aforementioned interfacial characteristics. In addition, the insets in Figure 6(b) indicate that the cracks in W/V composite mostly propagate along the interface, while for the W/VNb5 composite, cracks prefer going across the interface and propagate in the W fibers. This indirectly evidenced the better interfacial bonding due to the Nb addition.

In summary, the atomic interaction mechanism is effective in optimizing the interfacial characteristics of W/Zr-based BMGC and can be employed to guide the design of *in-situ* coating in many systems. Due to strong atomic interaction between W and Nb elements, the addition of Nb into the Zr-based alloy can result in the formation of Nb rich layer at the interface of the composite. The results agree with the calculation well and the segregation of Nb on W fiber surface effectively suppresses the formation of brittle W-Zr phase and the erosion of W fiber. The addition of Ta playing the similar role as Nb improves the interface as well. Nevertheless, Nb is more suitable to produce *in-situ* coating. Accordingly, the compressive and tensile properties of W fiber reinforced Zr-based BMGC are improved obviously. The tensile strength of the BMGC is increased more than 10%, which promotes the engineering application of the composite greatly.

## Methods

The alloys were prepared by arc melting the mixture of pure metals (purity is above 99.9%) under Ti-gettered argon atmosphere. W fibers with the diameter of 0.3 mm were provided by Xiamen Tungsten CO., Ltd. and corresponding tensile strength is about 2200 MPa. DSC analyze was carried out on Netzsch 404C at the heating rate of 20 K·min^−1^. The interface between Zr based alloys and W substrate was investigated using the sessile drop technique under 1273 K and 30 min in the vacuum of 5 × 10^−4^ Pa. BMGCs with the diameter of 8 mm were fabricated using the pressure infiltration technique. W fibers were precleaned by ethanol and then put into stainless steel tubes with close-packing. The samples for mechanical tests are all machined from the master BMGCs prepared under 1123 K and 5 min. Compressive and tensile samples are Φ5 × 10 mm and Φ5 × 20 mm (gauge length), respectively. Interfacial microstructure was examined using scanning electron microscopes (SEM, FEI Quanta 600 and ZEISS Supra 55) equipped with energy dispersive spectrometers (EDS) and transmission electron microscopy (TEM, FEI Tecnai F20 and Tecnai T12). The quasi-static compressive and tensile properties were characterized with Shimadzu AG-I 500 kN and Instron 5582 testing machines, respectively at a nominal strain rate of 5 × 10^−4^ s^−1^ at room temperature.

## Author Contributions

H.F.Z., H.M.F. and Z.Q.H. proposed the idea and designed the study. Z.K.L., A.M.W. and H.L. carried out the sessile drop experiments. H.M.F. fabricated the composites. Z.K.L. performed SEM, TEM and mechanical tests. Z.K.L. and P.F.S. derived and calculated the atomic interaction energy. H.F.Z., H.M.F., H.W.Z. and Z.W.Z. provided insightful discussions and writing suggestions for the manuscript.

## Supplementary Material

Supplementary Information Supplementary material for the corresponding SEM images at the interface in various systems

## Figures and Tables

**Table 1:** Segregated elements at the interface between W and alloy melts in various systems

Alloys	Segregated Elements
Zr_55_Cu_30_Ni_5_Al_10_^29^	Zra,^25^
Zr_52.25_Cu_28.5_Ni_4.75_Al_9.5_Ag_5_	Zr^a^
Zr_52.25_Cu_28.5_Fe_4.75_Al_9.5_Ag_5_	Zr^a^
Zr_52.25_Cu_28.5_Co_4.75_Al_9.5_Ag_5_	Zr^a^
Zr_52.5_Cu_17.9_Ni_14.6_Al_10_Ti_5_^30^,^31^,^32^	Zr^a^
Zr_57_Cu_15.4_Ni_12.6_Al_10_Nb_5_^30^,^33^,^34^	Nba,^25^
Zr_65_Cu_17.5_Ni_10_Al_7.5_^18^	Zr^18^
(Zr_55_Al_10_Ni_5_Cu_30_)_98_Nb_2_^25^	Nb^25^
Zr_47_Ti_13_Cu_11_Ni_10_Be_16_Nb_3_^25^	Nb^25^
Zr_40.08_Ti_13.30_Cu_11.84_Ni_10.07_Be_24.71_	Zr^a^
Zr_38.1_Ti_12.6_Cu_11.2_Ni_9.6_Be_23.5_Nb_5_	Nb^a^
Zr_38.1_Ti_12.6_Cu_11.2_Ni_9.6_Be_23.5_Ta_5_	Ta^a^
Zr_38.1_Ti_12.6_Cu_11.2_Ni_9.6_Be_23.5_Cr_5_	Zr^a^
Cu_1-x_Fe_x_ (0.4 ≤ x ≤ 1.6)^35^	Fe^35^
Cu_50_Zr_43_Al_7_^18^	Zr^18^
Cu_47_Ti_33_Zr_11_Ni_6_Sn_2_Si_1_^18^	Zr^18^

^a^The experimental results in this work.
