# Supplementary material for: Atomic interaction mechanism for designing the interface of W/Zr-based bulk metallic glass composites
Source: Sci Rep. 2015 Mar 11;5:8967. doi: 10.1038/srep08967 (PMC4355671; doi:10.1038/srep08967)
Supplement: Supplementary Information — Supplementary material for the corresponding SEM images at the interface in various systems [file srep08967-s1.pdf]

Atomic interaction mechanism for designing the interface of W/Zr-based bulk metallic glass composites

Z.K. Li[1,2], H.M. Fu[1], P.F. Sha[1,2], Z.W. Zhu[1], A.M. Wang[1], H. Li[1], H.W. Zhang[1], H.F. Zhang[1,*] , and Z.Q. Hu[1]

[1] *Shenyang National Laboratory for Materials Science, Institute of Metal Research, Chinese Academy of Sciences, Shenyang, 110016, China*

[2] *University of Chinese Academy of Sciences, Beijing, 100049, China*

[*] *Corresponding author: hfzhang@imr.ac.cn (H.F. Zhang)*

Fax: +86-24-23971783; Tel: +86-24-23971783

Supplementary material for the corresponding SEM images at the interface in various systems

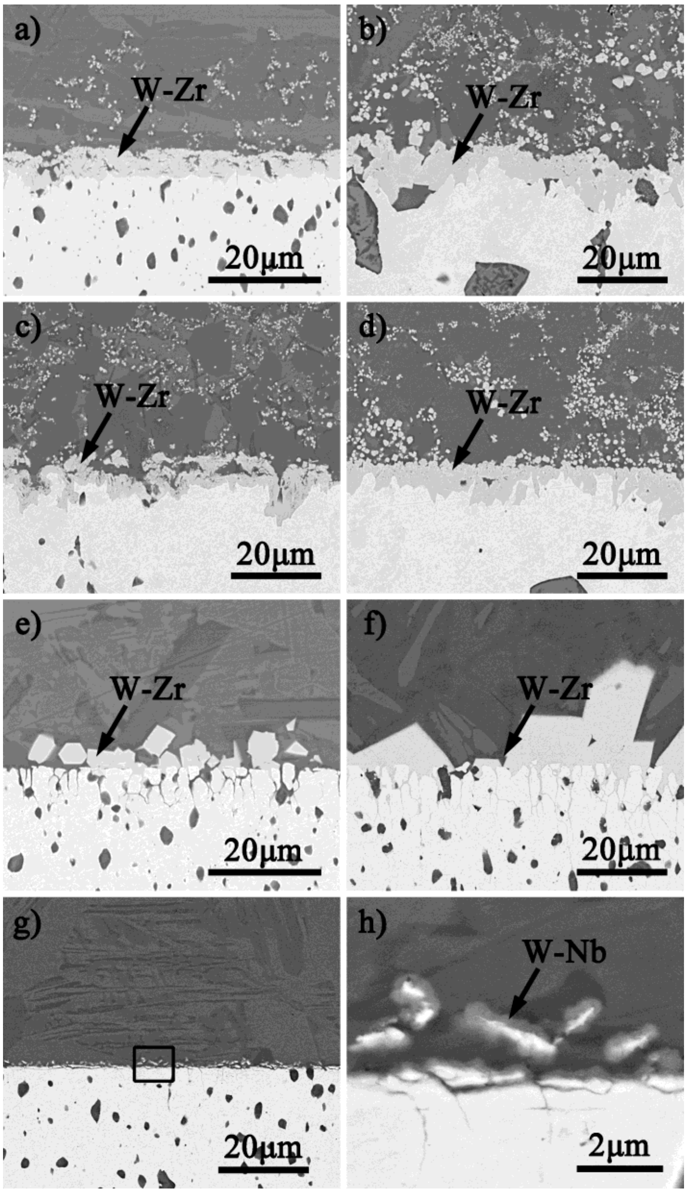

Figure S1 | SEM images at the interface between W substrate and a) $Zr_{55}Cu_{30}Ni_{5}Al_{10}$, b) $Zr_{52.25}Cu_{28.5}Ni_{4.75}Al_{9.5}Ag_{5}$, c) $Zr_{52.25}Cu_{28.5}Fe_{4.75}Al_{9.5}Ag_{5}$, d) $Zr_{52.25}Cu_{28.5}Co_{4.75}Al_{9.5}Ag_{5}$, e) $Zr_{52.5}Cu_{17.9}Ni_{14.6}Al_{10}Ti_{5}$, f) $Zr_{38.1}Ti_{12.6}Cu_{11.2}Ni_{9.6}Be_{23.5}Cr_{5}$ and g) $Zr_{57}Cu_{15.4}Ni_{12.6}Al_{10}Nb_{5}$, respectively. h) Enlarged view of the corresponding area in g).
